# Current Perspectives on Mesenchymal Dendritic Cell Neoplasms of Lymphoid Tissue: Insights into Ontogeny, Updates on Classification, and Clinicopathologic Characteristics

**DOI:** 10.3390/cancers17122055

**Published:** 2025-06-19

**Authors:** Neha Seth, Jithma P. Abeykoon, Gaurav Goyal, Ronald S. Go, Steven Tessier, Rebecca L. King, Aishwarya Ravindran

**Affiliations:** 1 Department of Pathology and Laboratory Medicine, Donald and Barbara Zucker School of Medicine at Hofstra/Northwell, New York, NY 11548, USA; 2 Division of Hematology, Department of Medicine, Mayo Clinic, Rochester, MN 55905, USA; 3 Division of Hematology/Medical Oncology, Department of Medicine, University of Alabama at Birmingham, Birmingham, AL 35233, USA; 4 Department of Internal Medicine, Mayo Clinic School of Graduate Medical Education, Rochester, MN 55905, USA; 5 Division of Hematopathology, Department of Laboratory Medicine and Pathology, Mayo Clinic, Rochester, MN 55905, USA; 6 Division of Laboratory Medicine-Hematopathology Section, Department of Pathology, University of Alabama at Birmingham, Birmingham, AL 35249, USA

**Keywords:** follicular dendritic cell, fibroblastic reticular cell, mesenchymal neoplasms, EBER-positive FDCS, EBER-positive FRCT

## Abstract

Mesenchymal dendritic cell neoplasms represent a distinct category of hematologic malignancies that challenge traditional classifications of histiocytic and classical dendritic/Langerhans cell neoplasms. Historically grouped under the broader umbrella of dendritic cell neoplasms, these entities differ significantly in their ontogeny, histopathologic features, molecular alterations, and clinical behavior. They are categorized into three main subtypes including follicular dendritic cell sarcoma, fibroblastic reticular cell tumor, and EBV-positive inflammatory follicular dendritic cell sarcoma/fibroblastic reticular cell tumor. They originate from mesenchymal stromal cells, and genetic alterations activating the NF- κβ pathway are frequent in follicular dendritic cell sarcomas. Immunophenotypic characterization is critical to distinguish these from other hematologic malignancies including histiocytic and classical dendritic/Langerhans cell neoplasms and other solid (non-hematopoietic) cancers. This review recapitulates current knowledge on existing classifications, details their diverse ontogeny from classical dendritic cell neoplasms, and provides insights into their clinicopathologic characteristics to improve diagnostic accuracy. We detail two case studies that demonstrate the challenges involved in the histopathologic diagnosis of these rare tumors, necessitating a comprehensive workup. Integrating developmental biology into practical diagnostic algorithms is essential to improve recognition and classification of these underdiagnosed neoplasms, ultimately guiding timely management.

## Simple Summary

Mesenchymal dendritic cell neoplasms are rare tumors that arise from stromal cells of lymphoid tissue, comprising three different subtypes including follicular dendritic cell sarcoma, fibroblastic reticular cell tumor, and EBV-positive inflammatory follicular dendritic cell sarcoma/fibroblastic reticular cell tumor. These tumors are biologically distinct from histiocytic and classical dendritic/Langerhans cell neoplasms that originate from hematopoietic precursors. The existing classifications of hematopoietic tumors create an overlap of these mesenchymal dendritic cell neoplasms with classical dendritic cell neoplasms and plasmacytoid dendritic cell neoplasms, resulting in diagnostic challenges in clinical practice. The purpose of this review is to provide insights into their ontogeny, detail their clinicopathologic features, and clarify their nomenclature in order to refine our understanding and enable accurate diagnosis of these entities.

## 1. Introduction

Hematopoietic neoplasms have historically been classified based on their presumed cell of origin, with the distinction between lymphoid, myeloid, plasma cell, and histiocytic/dendritic cell neoplasms being the central tenet of classification system [1,2]. Dendritic cell neoplasms, in particular, have long been grouped under histiocytic neoplasms due to their shared immune regulatory functions and antigen-presenting capabilities [3,4]. One of the main challenges in discussing follicular dendritic cell neoplasms is that the term “dendritic cell” is often misunderstood. It is a common misconception to associate dendritic cells with classical antigen-presenting dendritic cells that are ontogenically distinct from follicular dendritic cells. However, dendritic cell terminology has potentially resulted in the misclassification of follicular dendritic cell neoplasms within the spectrum of histiocytic/dendritic cell neoplasms.

Follicular dendritic cells (FDCs) and fibroblastic reticular cells (FRCs) are mesenchymal-derived stromal cells of lymphoid tissue and play key roles in the structural and functional organization of lymphoid tissues [5,6]. Their neoplastic counterparts include the following diseases: follicular dendritic cell sarcoma (FDCS), fibroblastic reticular cell tumor (FRCT), and Epstein–Barr virus (EBV)-positive inflammatory FDCS/FRCT. These FDC/FRC-derived tumors are biologically distinct from histiocytic and classical dendritic/Langerhans cell neoplasms which derive from hematopoietic precursors. Further, emerging data on genomic alterations in FDC neoplasms reveal distinct molecular pathways that set them apart from histiocytic and classical dendritic/Langerhans cell neoplasms [7,8,9]. As FDC/FRC-derived tumors originate from mesenchymal dendritic cells, the recent World Health Organization (WHO, fifth edition) has appropriately reclassified these entities under the umbrella of stroma-derived neoplasms of lymphoid tissue [10], which represents a change in classification category from the prior WHO (revised fourth edition, 2016), where these were classified under the category of histiocytic/dendritic cell neoplasms. The International Consensus Classification (ICC), however, continues to place these neoplasms on the spectrum of histiocytic/dendritic cell neoplasms, albeit acknowledging their mesenchymal derivation [11]. This discrepancy reflects a broader uncertainty in the field about how these tumors should be conceptualized.

The primary aim of this review is to detail the developmental biology of these mesenchymal dendritic cell neoplasms and their distinction from mononuclear phagocyte system, recapitulate the current knowledge on the existing classifications of hematopoietic tumors, and provide insights into the clinicopathologic characteristics of the various subtypes.

## 2. Ontogeny of Mesenchymal Dendritic Cells Versus the Mononuclear Phagocyte System

### 2.1. Ontogeny of Mesenchymal Dendritic Cells

**Figure 1:**
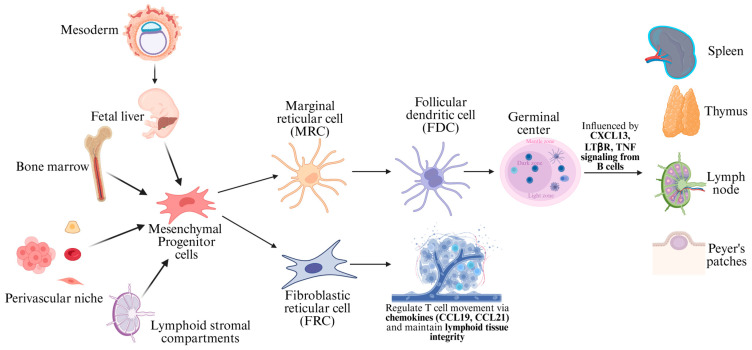
The ontogeny of the mesenchymal dendritic cells of lymphoid tissue illustrates the differentiation of follicular dendritic cells (FDCs) and fibroblastic reticular cells (FRCs) from mesenchymal progenitor cells. Mesenchymal progenitors arise from the fetal liver, bone marrow, perivascular niches, and lymphoid stromal compartments. A key transitional subset, the marginal reticular cell (MRC), arises from mesenchymal progenitors and gives rise to FDCs, influenced by CXCL13, lymphotoxin-β receptor (LTβR), and tumor necrosis factor (TNF) signaling from B-cells; they then localize to germinal centers and support B-cell selection and antigen retention in the lymph nodes, spleen, thymus, and Peyer’s patches. FRCs, on the other hand, differentiate directly from the mesenchymal progenitors to form the reticular network in T-cell zones, guiding T-cell migration via CCL19 and CCL21 while maintaining lymphoid tissue integrity.

Follicular dendritic cells and fibroblastic reticular cells are both specialized stromal cells found in secondary lymphoid organs. FRCs are also known as fibroblastic dendritic cells. Unlike classical dendritic cells that originate from hematopoietic stem cells (HSCs), both FDCs and FRCs are derived from mesenchymal stromal precursors, which are non-hematopoietic in origin and emerge during embryonic development in sites such as the fetal liver, bone marrow, perivascular niches, and lymphoid stromal compartments (Figure 1) [5,6,12]. These mesenchymal precursors give rise to lymphoid tissue organizer (LTo) cells which emerge during early embryogenesis in response to retinoic acid released by local neurons and represent an essential stromal cell population that lays the foundation for lymphoid organogenesis [6]. They begin expressing CXCL13 and engage in molecular crosstalk with lymphoid tissue inducer (LTi) cells. This interaction, largely mediated by lymphotoxin-β receptor (LTβR) signaling and the nuclear factor-kappa B (NF-κB) pathway, results in the formation of the lymph node anlage and leads to the emergence of FRC progenitors. These cells adopt a myofibroblastic phenotype, characterized by the expression of podoplanin (gp38), ER-TR7, and alpha-smooth muscle actin (α-SMA), and they differentiate into a heterogeneous network of mature FRCs [6].

FRCs predominantly reside in T-cell zones of lymphoid tissues including the lymph nodes, spleen, thymus, and Peyer’s patches, where they form an intricate three-dimensional stromal scaffold [6]. One of their key functions is the secretion of the homeostatic chemokines CCL19 and CCL21, which guide naïve T-cells and dendritic cells into the paracortex, promoting T-cell–DC interactions, which are crucial for adaptive immunity. They also maintain the structural integrity of lymphoid organs by producing extracellular matrix components and forming a conduit network that facilitates the rapid transport of soluble antigens and cytokines. Moreover, FRCs regulate immune tolerance by presenting peripheral tissue antigens in a non-inflammatory context and play a suppressive role during inflammation by limiting T-cell proliferation through mechanisms involving nitric oxide and prostaglandins [6,13].

FRCs are not a uniform population, and several functional subsets have been identified, each occupying distinct anatomical niches and playing specialized roles. T-zone reticular cells, located in the paracortex, produce CCL19 and CCL21 to guide T-cells and DCs [13]. Perivascular FRCs are closely associated with high endothelial venules (HEVs) and contribute to vascular barrier function via interactions with CLEC-2+ platelets. Medullary reticular cells, located in the medullary cords, create niches for plasma cell survival. B-cell zone reticular cells help to maintain follicular architecture and promote B-cell responses, particularly under chronic antigenic stimulation or after immunization [6,10]. A critical subset among these are the marginal reticular cells (MRCs), which are located beneath the subcapsular sinus. MRCs represent a transitional subset within the FRC lineage that has the capacity to give rise to FDCs [6,13,14]. During lymphoid tissue development and follicle formation, B-cell-derived signals, including LTβR and TNF, act on MRCs and promote their proliferation and differentiation into FDCs. This process is accompanied by continued production of CXCL13, which attracts CXCR5+ B-cells into forming follicles and establishes the microenvironment of the germinal center [14]. FDCs, once formed, settle in B-cell zones and play a central role in organizing germinal centers by presenting antigens in their native form to B-cells undergoing affinity maturation [15,16]. They express complement receptors CD21, CD23, and CD35, which allow them to trap immune complexes and retain them over long periods. These retained antigens provide a scaffold for competitive selection among germinal center B-cells, ensuring that only high-affinity clones survive to become plasma cells or memory B-cells [15,16,17]. Although morphologically dendritic, FDCs do not express MHC class II and do not process antigens for T-cell presentation. Instead, they function as long-lived antigen depots and key instructors of the humoral immune response.

Both FRCs and FDCs share a common mesenchymal ancestry but follow diverging differentiation paths shaped by their microenvironment. FRCs form the immunoregulatory and structural backbone of lymphoid tissues, while FDCs specialize in coordinating B-cell selection and survival within the germinal center [5,6,13]. Neoplasms originating from FDCs and FRCs parallel the phenotypes of their normal counterparts which aid in recognition and distinction from other malignant neoplasms.

### 2.2. Ontogeny of the Mononuclear Phagocyte System

**Figure 2:**
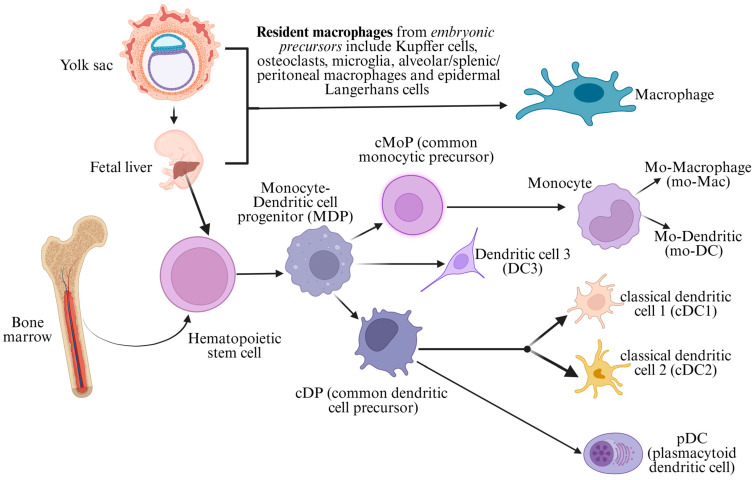
The ontogeny of the mononuclear phagocytic system illustrates the developmental pathways of monocytes, macrophages, and dendritic cells (DCs) from hematopoietic stem cells (HSCs) in the bone marrow and embryonic precursors. Resident macrophages originate from the yolk sac and fetal liver and are self-renewing. HSCs give rise to monocyte–dendritic cell progenitors (MDPs), which further differentiate into monocytes and dendritic cells. Monocytes generate monocyte-derived macrophages (mo-Macs) and monocyte-derived dendritic cells (mo-DCs), while common dendritic cell precursors (cDPs) give rise to plasmacytoid dendritic cells (pDCs) and classical (conventional) dendritic cells (cDC1 and cDC2), which are key antigen-presenting cells. This diagram highlights the dual origin of macrophages and dendritic cells, distinguishing self-renewing embryonic macrophages from bone marrow-derived immune cells.

The mononuclear phagocyte system comprises monocytes, macrophages, and dendritic cells that are derived from both embryonic and bone marrow-derived hematopoietic precursors (Figure 2) [18]. During embryogenesis, hematopoiesis occurs in the fetal yolk sac in the early stages (up to day 32 of gestation), and subsequently, the fetal liver takes over this function during the remainder of gestation [18,19]. At birth, the bone marrow becomes the principal site of hematopoiesis. However, there exists a unique physiology in the ontogeny of the resident macrophages (including epidermal Langerhans cells, osteoclasts, microglia, alveolar macrophages, splenic macrophages, peritoneal macrophages, and Kupffer cells), which are derived from embryonic precursors (the yolk sac and fetal liver) and are in a state of constant self-renewal that is independent of the bone marrow throughout post-natal life under steady-state conditions [19,20].

In the post-natal period, the HSCs give rise to monocyte–dendritic progenitors (MDPs), which then diverge into two main pathways: the common monocytic precursor (cMoP) and the common dendritic cell precursor (cDP) (Figure 2) [18,21,22,23]. The cMoP generates circulating monocytes, which can differentiate into tissue macrophages (mo-Macs) or monocyte-derived dendritic cells (mo-DCs) in response to inflammatory cues. Monocytes also retain the ability to give rise to Langerhans cells, especially during periods of skin inflammation or injury, underscoring the plasticity of monocyte-derived lineages [19]. Meanwhile, the cDP gives rise to pre-DCs, which exit the bone marrow, migrate to peripheral tissues, and differentiate into classical (conventional) dendritic cells (cDCs) and plasmacytoid dendritic cells (pDCs) [18,19,24].

Classical (conventional) dendritic cells include two major subtypes: cDC1 and cDC2 [21]. cDC1 cells, marked by CD141 in humans, specialize in cross-presenting antigens to CD8+ cytotoxic T-lymphocytes, playing a central role in antiviral and antitumor immunity. cDC2 cells, expressing CD1c and SIRPα, are adept at presenting antigens to CD4+ helper T-cells and directing T-cell polarization into Th1, Th2, or Th17 subsets [19,24]. Plasmacytoid dendritic cells are major producers of type I interferons and play an essential role in antiviral defense. Recently, a third population, termed DC3, has been described [25]. Unlike cDC1 and cDC2, which arise from the cDP lineage, DC3 cells appear to derive from MDPs. These cells share phenotypic features with both monocytes and cDC2 and become expanded under inflammatory conditions, infections, and cancers. Functionally, DC3 cells contribute to antigen presentation and cytokine secretion and are increasingly seen as a bridge population with both innate and adaptive characteristics [19,24,25]. The development of each of these dendritic cell subsets is regulated by lineage-specific transcription factors: IRF8 and BATF3 for cDC1, IRF4 for cDC2, and TCF4 (E2-2) for pDCs [22,26]. Monocyte differentiation into macrophages is guided by PU.1 and M-CSF, while mo-DCs often emerge under the influence of GM-CSF and IL-4 [18].

Neoplasms derived from this mononuclear phagocyte system are composed of histiocytic/macrophage neoplasms (including Rosai–Dorfman disease, Erdheim–Chester disease, ALK-positive histiocytosis, juvenile xanthogranuloma, and histiocytic sarcoma) and classical dendritic cell/Langerhans cell neoplasms (including indeterminate cell histiocytosis, interdigitating dendritic cell sarcoma, Langerhans cell histiocytosis, and Langerhans cell sarcoma), respectively [11,27,28,29]. Understanding the precise developmental trajectories of these cell types has become increasingly important, and ontogeny-informed classification systems may have implications for future diagnostic and therapeutic strategies.

## 3. Classification: Past, Present and Future

The classification of histiocytic/dendritic cell neoplasms has undergone significant refinements over time, reflecting advances in molecular genetics, immunophenotyping, and cell lineage tracing. Acknowledging the distinct biology and evolution of mesenchymal dendritic cell neoplasms, the recent fifth edition of the WHO has recognized FDCS, FRCT, and EBV-positive inflammatory FDCS under the distinct category of mesenchymal stromal neoplasms of lymphoid tissue and has moved them out of the category of histiocytic/dendritic cell neoplasms. Further, the current fifth edition of the WHO subclassifies the plasmacytoid dendritic cell neoplasms on the spectrum of histiocytic/dendritic cell neoplasms due to their ontogeny derivation from common dendritic cell progenitors (Figure 2) [10]. The ICC largely mirrors the revised fourth edition of WHO but incorporates terminology updates and slight re-organization of these entities [8]. A structured comparison between WHO (fourth edition), WHO (fifth edition), and ICC, highlighting changes in nomenclature, classification criteria, and newly recognized entities is detailed in Table 1.

In addition to the WHO and ICC frameworks, the revised Histiocyte Society (HS) Classification provides an alternative approach to categorizing histiocytic disorders, subclassifying them into five different groups (L, C, R, M, H) based on their clinical behavior, molecular alterations, and systemic involvement rather than strict histopathologic criteria (Figure S1) [27]. This revised HS classification, similar to the current fifth edition of the WHO classification, does not include mesenchymal dendritic cell neoplasms in the classification of histiocytosis given their distinction in ontogeny.

**Figure 3:**
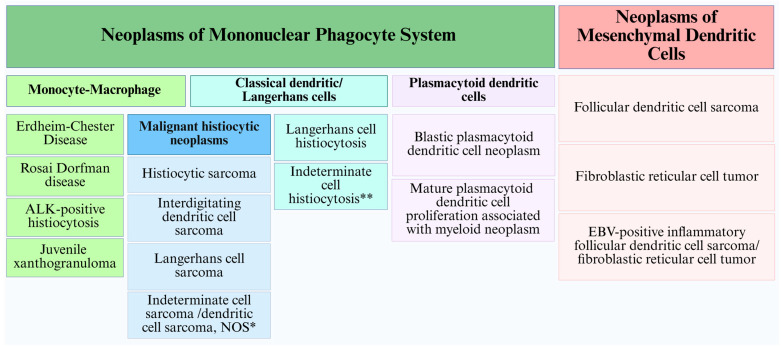
Categorization of histiocytic and various dendritic cell neoplasms according to their putative cell of origin. Abbreviations: NOS: not otherwise specified * Indeterminate cell sarcoma is a subtype recognized by the 2016 Revised Histiocyte Society Classification. The current WHO (fifth edition) and ICC classifications do not recognize this subcategory; therefore, this entity may be termed ‘dendritic cell sarcoma, NOS’ [11,27,28,29]. ** Indeterminate cell histiocytosis is synonymous with indeterminate dendritic cell tumor/indeterminate dendritic cell histiocytosis.

While the current classifications provide useful diagnostic frameworks, they do not fully account for the fundamental differences in cellular origin between histiocytic, classical dendritic cell, Langerhans cell, plasmacytoid dendritic cell, and mesenchymal dendritic cell neoplasms. A proposed outline of the categories of various dendritic cell neoplasms relative to their putative cell of origin is presented in Figure 3.

## 4. Clinical Features and Histopathologic Characterization of Mesenchymal Dendritic Cell Neoplasms

**Figure 4:**
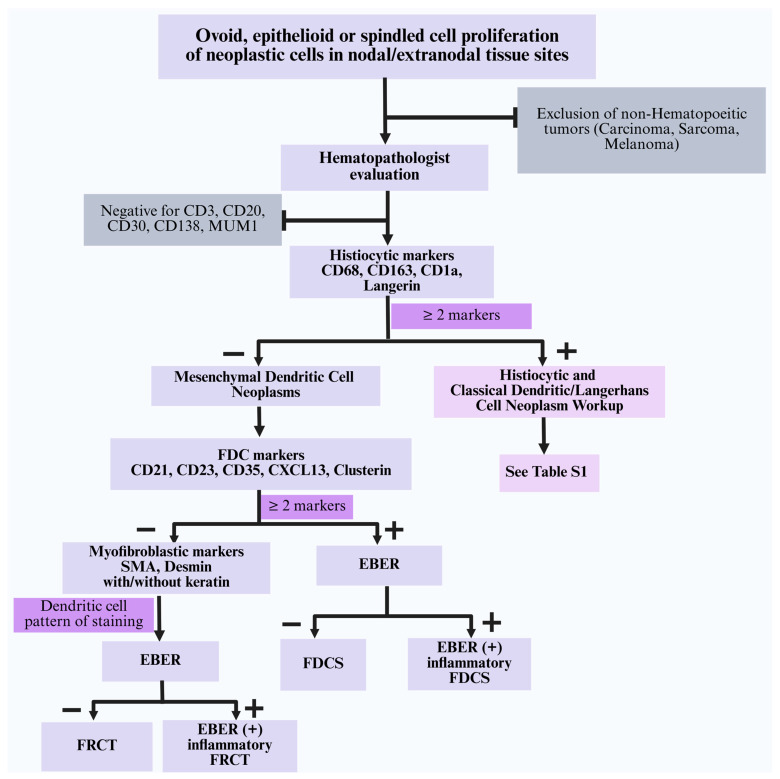
Algorithm for workup of mesenchymal dendritic cell neoplasms and distinction from histiocytic and classical dendritic/Langerhans cell neoplasms. Abbreviations: FDC: follicular dendritic cell; FDCS: follicular dendritic cell sarcoma; EBER: Epstein–Barr virus encoded RNA by in situ hybridization; FRCT: fibroblastic reticular cell tumor.

The diagnosis of mesenchymal dendritic cell neoplasms presents a unique challenge due to their morphologic overlap with other neoplasms, particularly histiocytic and classical dendritic/Langerhans cell neoplasms and other solid (non-hematopoietic) tumors. A structured immunohistochemical (IHC) approach is essential in order to accurately classify these tumors and distinguish them from other spindled cell/ epithelioid neoplasms. To streamline this diagnostic process, a stepwise IHC algorithm (Figure 4) outlining a systematic approach to evaluating ovoid/epithelioid or spindled cell proliferations with lymphoplasmacytic infiltration is described.

### 4.1. Follicular Dendritic Cell Sarcoma

#### 4.1.1. Clinical Features

FDCS accounts for <1% of all soft tissue sarcomas [30]. The Surveillance, Epidemiology, and End Results (SEER) program, which represents approximately 48% of the United States population across 22 national cancer registries, identified 368 FDCS cases between 2001 to 2022, corresponding to an age-adjusted incidence rate of 1.1 per 10 million individuals (95% confidence interval: 1.0–1.3) [31,32]. Alternate acceptable terminology for FDCS includes ‘follicular dendritic cell tumor’ [28,33]. FDCS involves both nodal and extranodal sites, and typically presents as a slow-growing, painless mass without associated B-symptoms (i.e., fever, night sweats, and weight loss). The median age at presentation is around 50 years with no sex predilection, and common sites of involvement include the gastrointestinal tract and retroperitoneum, also including the intra-abdominal lymph nodes [34]. Radiologic features are not pathognomonic and often overlap with other neoplasms including lymphomas and metastatic tumors [35]. Computed tomography (CT) scans of nodal sites present as well-delineated homogenous masses, whereas extranodal sites present as heterogeneous masses with or without internal necrosis and calcification [35]. A subset of cases presents in association with Castleman disease or autoimmune diseases [36,37].

#### 4.1.2. Histopathologic Features

On hematoxylin and eosin (H&E)-stained sections, FDCS is characterized by fascicular, whorled, syncytial, or storiform growth pattern of ovoid, epithelioid, or spindled cells with vesicular chromatin, small conspicuous nucleoli, and eosinophilic cytoplasm with indistinct cell borders [33]. These tumor cells are often binucleated, reminiscent of normal FDCs within germinal centers. They are associated with varying degrees of inflammatory cells in the background composed of predominantly small mature lymphocytes and plasma cells with occasionally admixed eosinophils or neutrophils.

By immunohistochemistry, the most sensitive FDC markers include CXCL13, CD21, and clusterin, whereas the most specific markers are CD21, CD23, CD35, and CXCL13 [33,38]. At least two of these five FDC markers need to be present for a diagnosis of FDCS. In addition, other positive IHC markers described in FDCS include podoplanin/D2-40, SSTR2a, follicular dendritic cell secreted protein (FDCSP), serglycin (SRGN), L1CAM, vimentin, and fascin [39,40,41,42]. Benign immature TdT-positive T-cell lymphoblastic proliferations are present in 40–50% of cases, and the abundance of these TdT-positive cells is usually associated with paraneoplastic manifestations [37]. FDCS is negative for histiocytic markers (CD163, CD14, CD1a, langerin, ), although CD68 expression can be variably present in a subset of tumor cells [43]. Other pertinent negative immunostains include hematolymphoid markers (CD45, CD3, CD20, CD79a), myoid markers (smooth muscle actin (SMA), desmin), vascular markers (CD31, CD34), epithelial markers (EMA, keratins), melanoma markers (melanA, SOX10, HMB45, S100), and other markers (ALK, CD30) [44,45]. PD-L1 expression can be present in a subset of FDCS and may have implications for immunotherapy [46].

#### 4.1.3. Follicular Dendritic Cell Sarcoma and Castleman Disease: Understanding the Connection

**Figure 5:**
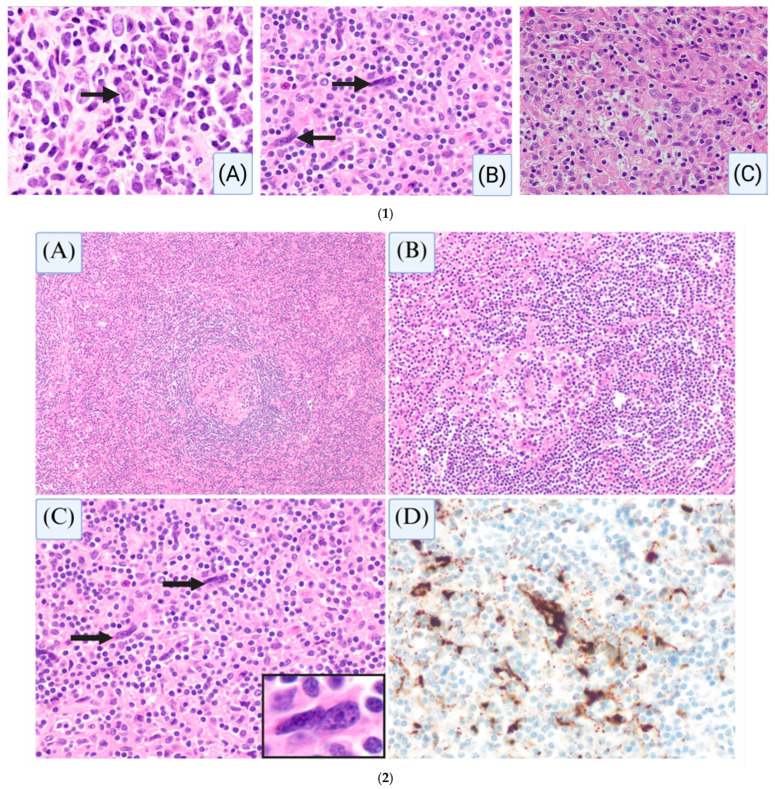
(**1**): Morphologic features of normal follicular dendritic cells, dysplastic follicular dendritic cells, and neoplastic follicular dendritic cells. Histologic sections demonstrate normal follicular dendritic cells ((**A**), arrow) within the germinal center of a reactive lymph node. Dysplastic follicular dendritic cells ((**B**), arrows) characterized by binucleation with hyperchromasia are present in hyaline vascular Castleman disease. Sheets of neoplastic follicular dendritic cells characterized by ovoid/epithelioid nuclei (**C**) in follicular dendritic cell sarcoma are also shown. Magnification (**A**–**C**) ×400. (**2**): Hyaline vascular variant of Castleman disease (HVCD) with dysplastic follicular dendritic cells. Hematoxylin and eosin (H&E)-stained sections show an atretic follicle ((**A**) ×40, (**B**) ×200) with concentric rings of mantle-zone B-cells (onion-skinning) with a hyalinized vessel penetrating the follicle (lollipop sign), associated with dysplastic follicular dendritic cells in the extrafollicular areas ((**C**) ×200, arrows) that show positive expression of CXCL13 ((**D**) ×200).

While its exact prevalence is unknown, there is increasing recognition of FDCS associated with Castleman disease, particularly the unicentric hyaline vascular subtype (HV-UCD) [47]. A hyperplasia–dysplasia–neoplasia model of FDC proliferation has been proposed for the link between HVCD and FDCS (Figure 5(1)). In this model, FDC hyperplasia occurs initially as a reactive proliferation in HV-UCD, characterized by tight concentric mantle zones and prominent vascularity [47]. Over time, a subset of these proliferating FDCs may acquire cytologic atypia—marked by nuclear enlargement, hyperchromasia, and multilobation—representing dysplasia (Figure 5(2)). These atypical or dysplastic FDCs may localize within the germinal centers or spill into the interfollicular/extrafollicular areas [48]. Studies have demonstrated shared clonality between dysplastic FDCs and FDCS as well as epidermal growth factor receptor (EGFR (HER1)) overexpression supporting a molecular continuum between hyperplastic and neoplastic states [49,50].

Dysplastic FDCs can be seen within follicles and sometimes in extrafollicular areas of HV-UCD, displaying enlarged, often binucleated, and occasionally multinucleated nuclei with hyperchromasia. These changes may mimic Reed–Sternberg-like cells, and caution should be exercised to avoid a misdiagnosis of classic Hodgkin lymphoma [51]. Critically, stroma-rich variants of HV-UCD exist, and a concurrent diagnosis of FDCS in HV-UCD should only be made in the setting of a distinct mass-like lesion composed of sheets of neoplastic FDCs.

### 4.2. Fibroblastic Reticular Cell Tumor (FRCT)

#### 4.2.1. Clinical Features

FRCT is a neoplasm derived from stromal fibroblastic reticular cells, and its precise incidence is unknown. Although the SEER program has been collecting FRCT data since 2010, only a single case has been reported thus far, highlighting the exceptional rarity of the disease [31]. Alternate acceptable terminologies may include ‘fibroblastic dendritic cell tumor’ and ‘fibroblastic reticular cell sarcoma’ [52]. FRCT typically presents as a localized disease, and nodal sites are more commonly involved than extranodal sites. The median age at presentation is 61 years with no definite sex predilection [34,52]. There are no characteristic radiologic findings described in FRCTs primarily due to the relative rarity of this tumor compared to FDCS.

#### 4.2.2. Histopathologic Features

On H&E-stained sections, FRCTs show similar morphologic features to FDCS, composed of ovoid/epithelioid to spindled cells with varying nuclear size, arranged in a whorled, fascicular, or storiform growth pattern and accompanied by inflammatory cells composed of predominantly lymphocytes and plasma cells. Intercellular collagen fibrils are often present.

By immunohistochemistry, there is considerable overlap with soft tissue sarcomas of mesenchymal origin, demonstrated by variable positivity for myoid markers (SMA and desmin) in a dendritic cell pattern that highlights the delicate cytoplasmic extensions of these FRCT cells [53]. Cytokeratin positivity may be present in up to two-thirds of cases, where cytoplasmic processes are highlighted, and caution must be exercised to not over interpret these as metastatic carcinomas with spindled morphology [53,54]. Cytokeratin-positive FRCTs were previously known as ‘cytokeratin-positive interstitial reticular cell tumor’, and these frequently co-express SMA with/without desmin [53]. Variable CD68 expression can be present in FRCT [55,56]. Other positive IHC markers include caldesmon, fascin, vimentin, and EMA [55]. The pertinent negative markers include histiocytic markers (CD163, CD14, CD1a, langerin), FDC markers (CD21, CD23, CD35, clusterin, CXCL13), hematolymphoid markers (CD45, CD3, CD20, CD79a), vascular markers (CD31, CD34), melanoma markers (melanA, SOX10, HMB45, S100), and others (ALK, CD30).

Ultrastructural examination of the FRCT is characterized by the presence of elongated/oval indented nuclei and slender cytoplasmic processes with a moderate amount of endoplasmic reticulum and intercellular collagen, often in close proximity to the lymphoid scaffold composed of small mature lymphocytes and plasma cells [57]. In the current era of modernized medical practice, electron microscopy for the evaluation of hematopoietic tumors is not widely applicable, thereby necessitating IHC workup. Diagnosing EBER-negative FRCT is particularly challenging due to its overlapping myoid/epithelial phenotypes, and therefore, this entity should essentially be a diagnosis of exclusion upon thorough evaluation of other non-hematopoietic tumors.

### 4.3. EBV-Positive Inflammatory FDCS/FRCT

All FDCS and FRCT cases should be evaluated for Epstein–Barr virus encoded RNA (EBER) by in situ hybridization studies and if positive should be classified as ‘EBV-positive inflammatory FDCS’ or ‘EBV-positive inflammatory FRCT,’ in accordance with concurrent IHC evaluation of FDC and myoid markers [58]. This entity was initially described as ‘EBV-positive inflammatory pseudotumor’ and showed positive expression of EBV latent membrane protein (LMP1) [59,60]. These cases are often incidental findings on imaging and occur almost exclusively in the liver and spleen, although other extranodal sites have been described on rare occasions [61]. These tumors show a rich inflammatory background composed of lymphocytes and plasma cells. Increased numbers of IgG4-positive plasma cells may also be present [58].

### 4.4. Overlap of FDCS and FRCT Morphologies

Occasional cases may demonstrate overlap of FDCS and FRCT immunophenotypes characterized by variable expression of FDC markers (CD21, CD23, CD35, CXCL13, clusterin) along with SMA and/or desmin with or without EBER-positivity [59,62]. In EBER-negative cases, they are more appropriately classified as ‘mesenchymal dendritic cell neoplasm with features indeterminate between FDCS and FRCT.’ In EBER-positive cases, they are best categorized as ‘EBV-positive inflammatory mesenchymal dendritic cell neoplasm with features indeterminate between FDCS and FRCT.’

### 4.5. Differential Diagnosis of Mesenchymal Dendritic Cell Neoplasms

(i) Histiocytic and classical dendritic/Langerhans cell neoplasms: Tumor cells in this category show expression of at least two monocyte/macrophage markers (CD68, CD163, CD4, CD14) or classical dendritic/Langerhans cell markers (CD1a, langerin). They are consistently negative for specific FDC markers (CD21, CD23, CD35, CXCL13), myoid markers (SMA, desmin), and EBER [63]. A summary of the morphologic and immunophenotypic characteristics of various subtypes is described in Table S1.
(ii) Blastic plasmacytoid dendritic cell neoplasm (BPDCN): This aggressive disease is considered an acute leukemia; thus, its clinical presentation should effectively distinguish this entity from the other lesions discussed herein. Histologically, BPDCN shows sheets of small to medium-sized cells with a uniform blast-like morphology, scant cytoplasm, and fine chromatin, often mimicking a myeloid sarcoma. BPDCN is positive for pDC markers (CD123, CD303, CD304, TCL1, TCF4), CD4, and CD56, and is negative for histiocytic and classical dendritic/Langerhans cell markers, FDC markers, SMA, desmin, and EBER [11,28]. Morphologically and clinically, these immature pDC-derived tumors are distinct from other histiocytic and classical dendritic/Langerhans cell neoplasms and are therefore excluded from the IHC algorithm (Figure 4).
(iii) Inflammatory myofibroblastic tumors (IMTs): IMTs show spindled or stellate cells with mixed inflammatory infiltrates. Tumor cells are positive for SMA and variably positive for desmin. Approximately 50–60% of cases exhibit cytoplasmic ALK expression due to *ALK* gene rearrangement. These cases are negative for FDC markers and EBER [52,64]. ALK-negative IMTs may be more challenging to distinguish from FRCT; in such cases, molecular analysis is helpful as IMTs may harbor *ROS1*, *NTRK*, *PDGFRβ*, and *RET* gene fusions, amongst others [64].
(iv) Kaposi sarcoma: The morphologic features of these sarcomas are characterized by slit-like vascular spaces with spindled endothelial proliferation that are positive for HHV8, D2-40, and vascular markers (CD31, CD34, ERG, FLI-1). These tumors lack SMA, desmin, FDC markers, and EBER.
(v) Metastatic carcinoma: These cases typically show nests or sheets of epithelioid cells with marked cytologic atypia and demonstrate uniform expression of keratins. The presence of true epithelial differentiation and absence of FDC or myoid markers exclude FDCS/FRCT.
(vi) Metastatic melanoma: These cases have varying morphologies composed of ovoid/epithelioid or spindled cells, often with prominent nucleoli; the cytoplasm may contain brown pigment (melanin). Immunostains are positive for ≥ 2 melanocytic markers (S100, SOX10, Melan A, HMB45). They are negative for FDC markers, SMA, desmin, and EBER.
(vii) Leiomyosarcoma: The histology of these sarcomas shows intersecting fascicles of spindled cells with cigar-shaped nuclei, varying degrees of nuclear pleomorphism, frequent mitoses, and areas of coagulative necrosis. Immunostains show diffuse SMA positivity and variable desmin and caldesmon expression [65]. These tumors are negative for FDC markers, helping to distinguish them from FDCS. However, distinction from FRCT may be challenging due to overlapping phenotypes (SMA+, variably desmin+) and thus require careful correlation with site(s) of involvement, including the absence of lymphoid scaffold characteristics, architectural patterns, and cytologic features (including lack of delicate cytoplasmic extensions on IHC). Hormone receptor positivity, if present, can be useful for distinguishing leiomyosarcoma (estrogen receptor+, progesterone receptor+) from FRCT.
(viii) Rhabdomyosarcoma: These cases show small round or elongated/spindled cells with variable skeletal muscle differentiation. IHC is positive for myogenic markers including myogenin, MyoD1, desmin, and muscle-specific actin. These tumors are negative for FDC markers and EBER. While there is some immunophenotypic overlap with FRCT (variably desmin+), the presence of positive markers of skeletal muscle differentiation (myogenin, myoD1) distinguishes these tumors from FRCT.
(ix) Undifferentiated sarcoma (pleomorphic sarcoma, undifferentiated): These tumors are composed of highly pleomorphic cells, including bizarre multinucleated, spindled, and epithelioid forms. These tumors may show variable and often focal SMA and desmin expression but lack a consistent immunophenotype. They are negative for FDC markers and EBER. Differentiation from FRCT requires correlation with cytomorphologic features, which appear to be more uniform in FRCT compared to the varying degree of nuclear pleomorphism in pleomorphic sarcomas.

### 4.6. Mesenchymal Dendritic Cell Neoplasms—Case Studies

The following section describes two case scenarios reflecting the algorithmic approach to diagnosing mesenchymal dendritic cell neoplasms.

#### 4.6.1. Case 1

**Figure 6:**
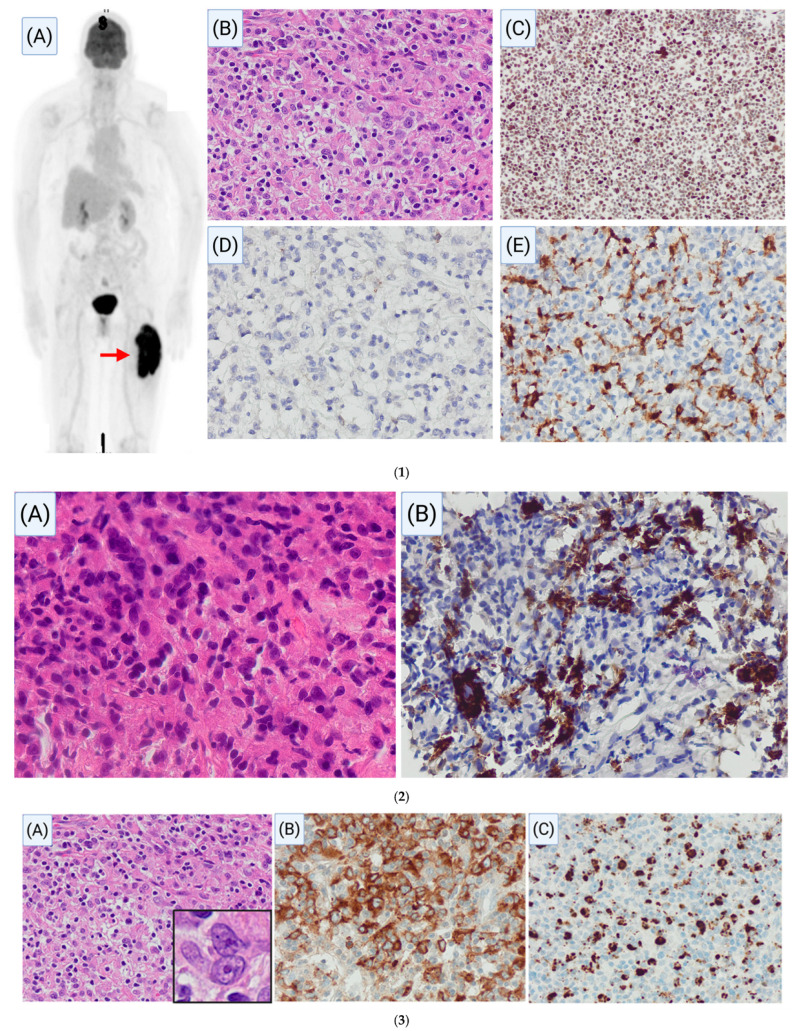
(**1**): Radiologic and histopathologic features of soft tissue thigh mass. (**A**) Whole-body PET-CT scan (skull vertex to mid-thigh) displaying a hypermetabolic mass (arrow) in the left thigh measuring 13 cm, with a standardized uptake value (SUV) of 16, indicative of a highly metabolically active lesion. Light microscopy of the soft tissue thigh mass ((**B**) hematoxylin and eosin, ×200) shows diffuse involvement by proliferation of epithelioid cells with indistinct cell borders which, by immunohistochemistry, retained BRG1 and INI-1 (**C**, ×200) expression and were negative for CD21 (**D**, ×200) and CD68 (**E**, ×200). CD68 highlights admixed reactive macrophages in the background. (**2**): Histopathology of lung biopsy. Light microscopy of lung biopsy demonstrates sheets of atypical epithelioid cells ((**A**) hematoxylin and eosin, ×600) that appear similar to the prior soft tissue thigh mass (Figure 6(1)), and it shows positive immunostaining for CD23 ((**B**) ×400) in a majority of tumor cells. (**3**): Re-evaluation of the soft tissue thigh mass. Light microscopy demonstrates sheets of atypical epithelioid cells ((**A**) hematoxylin and eosin, ×400; inset shows binucleated tumor cells reminiscent of normal cytology of follicular dendritic cells) that show positive immunostaining with clusterin ((**B**), ×400) and CXCL13 ((**C**), ×400).

A 71-year-old female with a past medical history of colon cancer, status-post hemicolectomy, presented with a six-month history of progressive left thigh swelling with no associated B-symptoms. A positron emission tomography–computed tomography (PET-CT) scan showed a hypermetabolic soft tissue mass in the left thigh measuring 13 cm in greatest dimension with a standardized uptake value (SUV) of 16, suggestive of a highly metabolically active process. Biopsy of the soft tissue mass revealed sheets of epithelioid cells with indistinct cell borders associated with background inflammatory infiltrates composed of small mature lymphocytes. An extensive IHC workup was performed and was unrevealing with negative staining for carcinoma (CAM5.2, pancytokeratin, CK7, CK20), melanoma (S100, SOX10, HMB-45, Melan-A), hematolymphoid markers (CD45, CD43, CD3, CD20, CD30, CD4), myeloid markers (CD34, CD117), myoid markers (SMA, desmin), neuroendocrine markers (CD56, synaptophysin, chromogranin, NSE), pDC markers (CD123, CD303), histiocytic markers (CD68, CD163, CD1a, langerin), and follicular dendritic cell marker (CD21). In addition, the tumor cells showed intact expression of BRG1 and INI-1, thus excluding the possibility of BRG1- and INI-1-deficient carcinomas. A comprehensive sarcoma fusion gene panel (including *ALK*, *CAMTA1*, *CCNB3*, *CIC*, *EPC1*, *EWSR1*, *FOXO1*, *FUS*, *GLI1*, *HMGA2*, *JAZF1*, *MEAF6*, *MKL2*, *NCOA2*, *NOTCH2*, *NTRK3*, *NUTM1*, *PDGFB*, *PLG1*, *ROS1*, *SS18*, *STAT6*, *TAF15*, *TCF12*, *TFE3*, *TRG*, *USP6*, and *YWHAE*) was negative for gene fusions. Taken together, this was initially classified as a high-grade malignant neoplasm, not further classifiable (Figure 6(1)).

The patient underwent radiation therapy to the left thigh followed by surgical resection of the residual tumor. Histopathologic examination of the excised lesion revealed an 11.5 cm fibrotic mass with focal necrosis but no viable tumor, suggesting a favorable response to treatment. At 5 months post-diagnosis, a chest-CT revealed increasing pulmonary nodules, with the largest nodule increasing in size from 18.5 mm to 19.3 mm, raising concern for pulmonary metastases. At 9 months post-diagnosis, ultrasound imaging also identified an enlarged left external iliac and common iliac lymph nodes measuring 3.5 × 3.1 cm, suggestive of recurrent disease. Biopsy of the lung (Figure 6(2)) revealed similar morphologic features to the thigh mass; however, at this point, additional immunostaining for CD23 was performed and demonstrated diffuse positivity in the tumor cells which triggered re-evaluation of the soft tissue thigh mass with additional FDC markers such as clusterin and CXCL13, which showed diffuse positivity in the tumor cells (Figure 6(3)). Subsequently, a diagnosis of follicular dendritic cell sarcoma was made at approximately 9 months from initial presentation.

This case highlights the atypical phenotype of FDCS with CD21-negativity and emphasizes the need for at least ≥ 2 FDC markers for a thorough histopathologic workup. Next-generation sequencing was performed on formalin-fixed paraffin-embedded tissue sections of the soft tissue thigh mass and revealed pathogenic mutations involving *RB1*, *TP53*, *PTEN*, and *KMT2A* genes.

#### 4.6.2. Case 2

**Figure 7:**
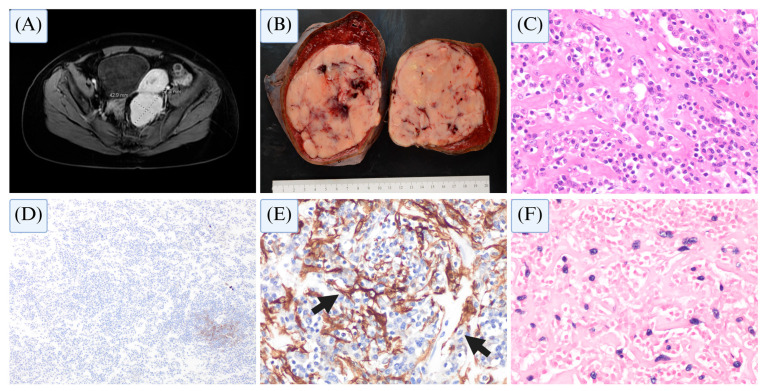
Radiologic and histopathologic features of splenic mass. (**A**) MRI abdomen (axial view) showing a large splenic mass, with a central bright T2 signal and an isointense peripheral stroma. (**B**) Gross pathology of splenic mass revealing a well-circumscribed, tan-white mass with areas of hemorrhage and focal necrosis, abutting the splenic capsule. (**C**) Hematoxylin and eosin (H&E, ×400) staining showing sheets of plump, ovoid cells with delicate cytoplasmic extensions and associated intercellular collagen fibers; these lesions cells are negative for CD21 ((**D**), ×100) and show positive expression of SMA ((**E**), ×400, arrows indicate the delicate cytoplasmic extensions) and EBER ((**F**), ×400).

A 63-year-old male with a history of hypertension presented for evaluation of a right renal mass which raised concerns about renal cell carcinoma. Contrast-enhanced magnetic resonance imaging (MRI) of the abdomen revealed two large masses: a right renal mass measuring 7.5 × 5.8 cm and a large splenic mass measuring 10 × 11.9 cm. The latter demonstrated a central bright T2 signal with an isointense peripheral stroma, raising suspicion for metastatic renal cell carcinoma (Figure 7A). The patient subsequently underwent partial right nephrectomy and splenectomy.

Histologic examination of the kidney mass confirmed chromophobe renal cell carcinoma. Gross examination of the splenic mass revealed a well-circumscribed, tan-white mass measuring 17.2 cm in its greatest dimension (Figure 7B), with scattered areas of hemorrhage and focal necrosis abutting the capsular surface. Microscopically, the splenic mass was composed of sheets of plump, ovoid cells with delicate cytoplasmic extensions (Figure 7C). Immunohistochemical staining demonstrated diffuse positivity for EBER and SMA, supporting an EBV-driven neoplastic process (Figure 7E,F). Cytologic features on SMA immunostaining highlighted the characteristic delicate cytoplasmic extensions seen in FRCT (Figure 7E). The neoplastic cells were negative for FDC markers (CD21, CD23; Figure 7D), pancytokeratin, desmin, myogenin, and MyoD1. The overall histopathologic findings were diagnostic of an EBV-positive inflammatory fibroblastic reticular cell tumor. The patient has remained clinically stable, with no evidence of disease recurrence at three years of follow-up.

This case highlights the typical indolent presentation of EBV-positive mesenchymal dendritic cell neoplasms that occur predominantly in the spleen and/or liver and may be identified incidentally during histopathologic workup of other solid tumors.

## 5. Mutational Landscape of Mesenchymal Dendritic Cell Neoplasms

The molecular pathogenesis of FDCS is characterized by recurrent genetic alterations that converge on dysregulation of the NF-κB signaling pathway. These include inactivating mutations in key NF-κB regulatory genes such as *NFKBIA*, *CYLD*, *BIRC3*, *SOCS3*, *TRAF3*, and *TNFAIP3*, leading to constitutive pathway activation. In addition, FDCS has alterations in tumor suppressor genes such as *CDKN2A*, *RB1*, and *TP53*, resulting in cell cycle deregulation and genomic instability. Mutations in immune evasion-related genes including *CD274* (PD-L1) and *PDCD1LG2* (PD-L2) have also been described, suggesting a potential mechanism for immune escape [9]. Recurrent mutations in tumor suppressors (*SETD2)* and chromatin modifiers *(CABIN1*, *NCAPH)* may lead to a disrupted epigenetic landscape promoting tumor progression through transcriptional dysregulation and chromatin misfolding [66]. Other genetic alterations, including copy number variations, somatic mutations involving oncogenes (*ZBTB7A*), and less commonly mutated genes (*BRAF V600E*, *PDGFRβ*), have been reported [7,66].

The mutational landscape of FDCS is biologically distinct from histiocytic and classical dendritic/Langerhans cell neoplasms, reflecting their differences in pathogenesis and oncogenic signaling pathways. While FDCSs frequently harbor genetic mutations activating the NF-κB pathway, histiocytic and classical dendritic/Langerhans cell neoplasms are predominantly characterized by mutations in the MAPK-ERK pathway (e.g., *BRAF*, *MAP2K1*, *KRAS*, *NRAS*) in approximately two-thirds of the cases [8], further validating the biological distinction amongst these entities [10]. Unlike FDCS, the genomic landscape of FRCT remains largely unknown due to its rarity and morphological overlap with soft tissue sarcomas. No recurrent genetic alterations or characteristic fusions have been described to date in FRCTs.

EBV-associated inflammatory FDCS/FRCT represents a rare variant with unique clinical and molecular features showing enrichment of the cAMP signaling pathway and can occasionally harbor a *STAT3* gene mutation within the SH2 domain resulting in increased protein phosphorylation and enhanced growth activity, suggesting a potential driver in tumorigenesis [58]. Unlike EBV-negative FDCS, EBV-positive FDCS lacks frequent NF-κB pathway mutations, highlighting a distinct pathogenic mechanism. Larger cohort studies are needed to systematically investigate the distinction in the genomic landscape of EBV-positive inflammatory FDCS/FRCTs from EBV-negative FDCS/FRCTs. Understanding these distinct genetic signatures is crucial for accurate diagnosis, prognostication, and the development of targeted therapies for these rare neoplasms.

## 6. Prognosis and Treatment Outcomes

### 6.1. Prognosis

The clinical course and prognosis of these neoplasms depend on tumor location, histologic features, molecular alterations, and response to therapy. While most mesenchymal dendritic cell neoplasms including EBV-positive cases exhibit an indolent course, a subset of them behave aggressively with recurrence and metastasis [34,53,67]. The prognosis of these tumors depends on several factors. Tumors smaller than 5 cm with a low mitotic index (<5 per HPF) and without necrosis tend to follow a more indolent course, while those with high mitotic activity (>5–10 per HPF), tumor size >5 cm, and necrosis are associated with higher recurrence and metastasis [34,35]. Metastases occur in 30–50% of FDCSs and 30–40% of FRCTs, with common sites including the lungs, liver, and intra-abdominal lymph nodes [53,68,69].

According to a study analyzing 66 patients with FDCS, the median overall survival (OS) following frontline therapy was 50 months [70]. The 5-year OS rate from a large cohort of 82 extranodal FDCS patients was reported to be 70%, with 2-year tumor-free OS rate estimated to be 68%; however, the 5-year tumor-free OS rate was estimated to be 32%, accounting for the likelihood of tumor recurrence [71]. A retrospective analysis from the SEER database indicated that patients with localized FDCS who underwent surgery had a 5-year OS rate of 88.4%, while those who received adjuvant therapy (chemotherapy or radiation versus both) along with surgery had a lower 5-year OS rate (68.4%), attributable to the disease burden at presentation [72]. Although FRCT shows predominantly similar survival rates to FDCS, data on FRCTs is limited, attesting to the extreme rarity of this tumor compared to other dendritic cell neoplasms [34,53].

### 6.2. Treatment

Treatment protocols in mesenchymal dendritic cell neoplasms are not standardized due to their low incidence and diverse biological behavior, resulting in a significant lack of consensus on treatment guidelines and best practices. The mainstay of treatment for localized FDCS and FRCT is surgical resection, with complete surgical excision or gross total resection (GTR) being essential for the optimization of progression-free survival (PFS) and OS [70,72,73]. Adjuvant radiotherapy may be considered post-surgery to enhance local control, PFS, and OS, particularly when GTR is achieved [53,72].

For metastatic or advanced tumors, systemic therapies are employed due to the absence of standard treatment protocols. Chemotherapy regimens, such as the combination of gemcitabine with a taxane, were associated with an overall response rate of 80% in 10 FDCS patients with measurable disease [70]. Lymphoma-based regimens such as CHOP (cyclophosphamide, hydroxydaunorubicin (doxorubicin), oncovin (vincristine), and prednisone), ICE (ifosfamide and etoposide+/− carboplatin), and ABVD (adriamycin (doxorubicin), bleomycin, vinblastine and dacarbazine) have been employed in disseminated tumors; however, data on outcomes are lacking [34,74]. Response to salvage therapy after recurrence remains poor, with the pattern of recurrence being predominantly locoregional [70]. Immune checkpoint inhibitors (ICIs) like pembrolizumab and PD-1 inhibitors such as sintilimab show potential when combined with chemotherapy. These combinations have been reported to achieve partial responses and a PFS of up to 17 months in rare cases [75]. Although FDCS can show high PD-L1 expression, the degree of response to ICI and its association with tumor PD-L1 expression is not well known [46,75]. This suggests that while no universal treatment protocol exists for advanced mesenchymal dendritic cell neoplasms, systemic therapies offer viable options.

Although rare, the identification of targetable mutations (such as *BRAF* V600E) can offer meaningful opportunities for integrating targeted therapies into treatment [9]. BRAF/MEK inhibitors provide a tailored therapeutic approach, potentially improving response rates and outcomes when conventional treatments fail. The use of these inhibitors, although contingent on the presence of specific mutations, represents a hopeful advancement in the treatment landscape of FDCS, underscoring the importance of genetic profiling in guiding therapeutic decisions [9].

Small molecular inhibitors have emerged as a potential treatment modality in these mesenchymal dendritic cell neoplasms. For instance, apatinib, an anti-angiogenic agent, exhibited favorable outcomes in a FDCS patient, resulting in a ten-month PFS after treatment [76]. Similarly, lenvatinib, a multi-kinase inhibitor targeting several receptor tyrosine kinases, achieved a seven-month PFS when combined with sintilimab in a patient with recurrent intestinal FDCS [77]. Furthermore, EGFR antagonists like cetuximab and panitumumab have shown in vitro effects on FDCS cells, highlighting the potential role these agents could play in targeting the EGFR, which is strongly expressed in FDCS [50].

In summary, the treatment landscape for FDCS and FRCT remains inadequately developed, and current management strategies with relative efficacy are detailed in Table 2. This absence of standardized care can lead to varied treatment approaches and outcomes across different institutions, reflecting the need for collaborative efforts among oncologists, researchers, and investigator-initiated clinical trials. Establishing precise treatment guidelines is essential for optimizing patient management and ensuring that individuals with mesenchymal dendritic cell neoplasms receive the most effective interventions available. Furthermore, ongoing research efforts are crucial to uncover novel therapeutic modalities, understand the underlying biology of these tumors, and identify potential biomarkers for targeted therapies. Future studies should focus on prospective clinical trials and international collaborations to establish evidence-based recommendations that can enhance treatment efficacy and improve quality of life for patients affected by FDCS and similar rare malignancies. If possible, participating in clinical trials should be encouraged for patients with FDCS and related conditions, as these trials provide access to cutting-edge therapies and contribute valuable data that can further advance the understanding and treatment of these challenging diseases.

## 7. Conclusions

Mesenchymal dendritic cell neoplasms differ significantly from histiocytic and classical dendritic/Langerhans cell neoplasms in their developmental origin, histopathologic characteristics, and genomic landscape. Their clinical behavior is usually indolent, but some cases can show an aggressive clinical course, with potential for recurrence and metastasis. As classification systems evolve, recognizing these distinctions is crucial for developing a more accurate taxonomy that reflects their true biology. Given the rarity of these neoplasms, there is a lack of standardized management guidelines, necessitating global collaborations. A precise distinction in nomenclature is crucial to eliminate ambiguities in disease recognition, optimize clinical decision making, and foster further research into targeted therapies for these rare but clinically significant neoplasms.

## Figures and Tables

**Table 1:** Distinction in classifications of mesenchymal dendritic cell neoplasms versus histiocytic and classical dendritic/Langerhans cell and plasmacytoid dendritic cell neoplasms: comparisons of the WHO (revised fourth edition and fifth edition) and ICC classifications of hematopoietic tumors.

WHO (Revised Fourth Edition, 2016)	WHO (Fifth Edition, 2022)	ICC (2022)
Follicular dendritic cell sarcoma Fibroblastic reticular cell tumor Inflammatory pseudotumor-like follicular/fibroblastic dendritic cell sarcoma Langerhans cell histiocytosis Langerhans cell sarcoma Indeterminate dendritic cell tumor Interdigitating dendritic cell sarcoma Erdheim–Chester disease Disseminated JXG Histiocytic sarcoma	**Mesenchymal Dendritic Cell** **Neoplasms** Follicular dendritic cell sarcoma Fibroblastic reticular cell tumor EBV-positive inflammatory FDCS ^#^ **Histiocytic/Dendritic Cell** **Neoplasms** Blastic plasmacytoid dendritic cell neoplasm Mature plasmacytoid dendritic cell proliferation associated with myeloid neoplasm * Langerhans cell histiocytosis Langerhans cell sarcoma Indeterminate dendritic cell tumor Interdigitating dendritic cell sarcoma Erdheim–Chester disease JXG Histiocytic sarcoma Rosai–Dorfman disease * ALK-positive histiocytosis *	Follicular dendritic cell sarcoma Fibroblastic reticular cell tumor EBV-positive inflammatory FDCS/FRCT Langerhans cell histiocytosis Langerhans cell sarcoma Indeterminate dendritic cell histiocytosis Interdigitating dendritic cell sarcoma Erdheim-Chester disease Disseminated JXG Histiocytic sarcoma Rosai-Dorfman disease * ALK-positive histiocytosis *

^#^ WHO (fifth edition) classification indicates fibroblastic reticular cell differentiation in EBV-positive mesenchymal dendritic cell neoplasms with lack of FDC markers, although it does not indicate EBV-positive FRCT as a distinct subtype but rather subclassifies within the category of EBV-positive inflammatory FDCS. * Newly recognized distinct neoplasms. Abbreviations: FDCS: follicular dendritic cell sarcoma; FRCT: fibroblastic reticular cell tumor; JXG: juvenile xanthogranuloma; WHO: World Health Organization; ICC: International Consensus Classification.

**Table 2:** Summary of current treatment modalities and their relative efficacy.

Treatment Modality	Description	Relative Efficacy
**Surgery**	Mainstay of treatment for localized disease	Complete surgical excision optimizes progression-free survival (PFS) and overall survival (OS) [70,72,73]
**Adjuvant Radiotherapy**	Considered post-surgery	Improves local control, PFS, and OS, especially after gross total resection
**Systemic** **Chemotherapy**	Used for metastatic or advanced tumors due to the lack of standard protocols	Higher overall response rates (~80%) in gemcitabine-based regimens in FDCS [34,70,74] Inadequate data on lymphoma-based regimens (CHOP, ICE, ABVD)
**Immune Checkpoint** **Inhibitors**	Pembrolizumab and other PD-1 inhibitors show potential when combined with chemotherapy	Potential for durable responses, especially in tumors with PD-L1 expression [46,75]
**Targeted Therapies**	Rare cases with MAPK pathway alterations may be amenable to treatment with BRAF/MEK-inhibitors	Offers tailored treatment with potential for improved outcomes [9]
**Small Molecule** **Inhibitors**	Apatinib (anti-angiogenic) and lenvatinib (multi-kinase inhibitor) have shown promise in individual cases	Limited data; additional research is required to evaluate long-term outcomes [76,77]

Abbreviations: CHOP: cyclophosphamide, hydroxydaunorubicin (doxorubicin), oncovin (vincristine) and prednisone; ICE: ifosfamide, carboplatin and etoposide; ABVD: adriamycin (doxorubicin), bleomycin, vinblastine and dacarbazine.

## Data Availability

Not applicable.

## Supplementary Materials

The following supporting information can be downloaded at: https://www.mdpi.com/article/10.3390/cancers17122055/s1. Figure S1: Revised classification of histiocytic disorders (including monocyte–macrophage and classical dendritic/Langerhans cell neoplasms) according to the Histiocyte Society (2016). Table S1: Histopathologic differences amongst subtypes of histiocytic and classical dendritic/Langerhans cell neoplasms.
