# Supplementary material for: Current Perspectives on Mesenchymal Dendritic Cell Neoplasms of Lymphoid Tissue: Insights into Ontogeny, Updates on Classification, and Clinicopathologic Characteristics
Source: Cancers (Basel). 2025 Jun 19;17(12):2055. doi: 10.3390/cancers17122055 (PMC12191123; doi:10.3390/cancers17122055)
Supplement: Supplementary file 1 [file cancers-17-02055-s001.zip › cancers-3672879-supplementary.pdf]

Figure S1: Revised Classification of Histiocytic disorders (including monocyte-macrophage and classical dendritic/Langerhans cell neoplasms) by the Histiocyte Society (2016)

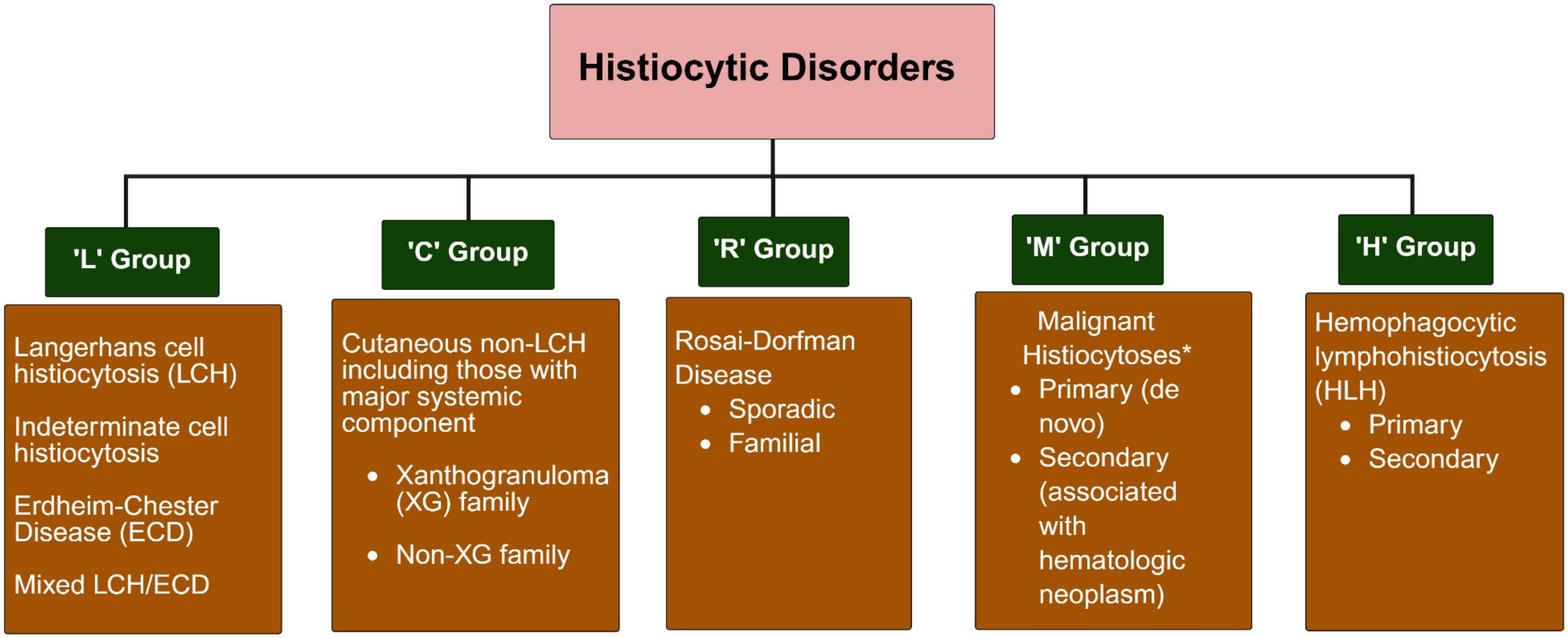

*Malignant Histiocytic Neoplasms is an umbrella term utilized by the Histiocyte Society and encompasses the following subtypes: histiocytic sarcoma, interdigitating dendritic cell sarcoma, indeterminate cell sarcoma, and Langerhans cell sarcoma [27, 63]

Table S1: Histopathologic Differences amongst Subtypes of Histiocytic and Classical Dendritic/Langerhans Cell Neoplasms

	Histiocyte/Macrophage Neoplasms				Classical Dendritic/Langerhans Cell Neoplasms		Malignant Histiocytic Neoplasms with Overlap of Monocyte-Macrophage and Classical Dendritic/Langerhans Cell Phenotypes[**]			
	**ECD[##]**	**JXG[##]**	**RDD**	**ALK+ histiocytosis**	**LCH**	**ICH**	**HS**	**IDCS**	**LCS**	**ICS/DCS, NOS**
Morphology	Bland-appearing histiocytes with abundant foamy cytoplasm and admixed Touton-type giant cells; Fibrosis and emperipolesis may be present	Similar to ECD morphology	Enlarged round to oval histiocytes with conspicuous nucleoli, pale chromatin, abundant pale eosinophilic / occasionally foamy cytoplasm with frequent emperipolesis	Varying morphologies: round to ovoid/epithelioid or spindled histiocytes with pale chromatin, variably district nucleoli, and moderate to abundant cytoplasm; emperipolesis may be present.	Oval histiocytes with folded/grooved nuclei, pale chromatin, inconspicuous nucleoli; moderate to abundant eosinophilic cytoplasm in a background of admixed	Morphological overlap with LCH	Pleomorphic histiocytes with overlapping morphologies with other poorly differentiated malignancies, including carcinoma, sarcoma, melanoma and anaplastic lymphomas			

				Possible morphologic overlap with RDD and ECD	eosinophils and small lymphocytes					
**Immunohistochemistry**										
CD68	+	+	+	+	+ (occasionally dot-like Golgi staining)	+ (occasionally dot-like Golgi staining)	+	+	+ (dot-like Golgi staining)	+ (dot-like Golgi staining)
CyclinD1	+/-	+/-	+	+	+	+	+	+	+	+
CD163	+	+	+/-	+	-/+	-/+	+	+/-	+/-	-/+
S100	-/+	-/+	+	+/-	+	+	-/+	+ (diffuse strong)	+	+
OCT2	-	-/+	+	+/-	-/+	-/+	-/+	+/-	-/+	-/+
CD1a	-	-	-	-	+	+	-	-	+	+
Langerin	-	-	-	-	+	-	-	-	+	-
ALK	-	-	-	+ (cytoplasmic/occasionally membranous or dot-like Golgi	-	-	-	-	-	-

				staining; never nuclear)						

Abbreviations: ECD: Erdheim-Chester disease; JXG: Juvenile xanthogranuloma; RDD: Rosai-Dorfman disease; LCH: Langerhans cell histiocytosis; ICH: Indeterminate cell histiocytosis; HS: Histiocytic sarcoma; IDCS: Interdigitating dendritic cell sarcoma; LCS: Langerhans cell sarcoma; ICS/DCS, NOS: Indeterminate cell sarcoma/Dendritic cell sarcoma, not otherwise specified.

[##]JXG and ECD have overlapping histopathologic features, and are distinguished by clinical and radiologic correlation [11, 27, 28]

[**]Malignant histiocytic neoplasms have overlapping features across monocyte/macrophage and classical dendritic/Langerhans cell phenotypes [63]

+/-: Positive in a majority of cases (>50%); negative in a minority

-/+: Negative in a majority of cases (>50%); positive in a minority
